# Health Tract, No. 165

**Published:** 1865

**Authors:** 


### HEALTH TRACT, No. 165.

### OBSCURE DISEASES.

Sometimes a physician is called to see a member of a family, who does not seem to be very sick, nor has become suddenly ill; the ailment appears to have manifested itself very gradually, and with all the powers of observation and comparison no adequate cause can be discovered. No one symptom is predominant in some cases; in others, the combination of symptoms and their character and quality are not like those usually observed. In cases like these, the physician is thrown upon his own resources, and employs remedial means on certain well-established general principles; but without any favorable result; the patient lingers still; then other principles and other remedies are applied, with no more encouraging results. Finally a change of air is advised in the shape of a visit to the country, to the sea-shore, or to the mountains, when the symptoms begin to abate; the patient regains accustomed health and vigor, and returns home reïnvigorated to a surprising degree; but, in a short time the old symptoms begin to return, and eventually acquire all their old power over the system.

Sometimes several members of a family are affected in the same way in the main; at others, only a single individual suffers. The reason for this is simply, that some persons are much more sensitive to the causes of disease than others; their constitutions are more susceptible of hurtful impressions. A practical inference may be very legitimately drawn from these statements, which, if heeded, would save many a valuable life in the course of any single year. The rule should be, when a person does not get better under the treatment of a skillful physician, in stead of wasting time, and endangering the permanent loss of health, and even life itself, to remove some distance from the locality. Or if a family seems to enjoy good health, and yet a servant or guest comes to remain several days or weeks, but is sure to get sick, the inference is the same, that there is some pernicious agency at work; so long so in the latter case, that the family have become habituated to it, while the stranger falls under its baleful influence. The wife may suffer and not the husband, because he may spend the larger part of his time from home; or being of a more delicate constitution, she is more impressible by delicate causes. There may be an unknown covered well or sink, or "fill up," under the house; some house-drain may be clogged up, or be broken in; some altera tions may have been made in improving or repairing the premises, or new slop-holes formed. A new kind of wall-paper may have been used in a particular room; lead water-pipes may have been recently introduced into the house, or may have been injured so as to detain water long enough to cause decomposition. For these reasons, some persons have better health in moving into other houses, on leaving their old ones, and *vice versa.*

